# Integration of RNAi and RNA-seq Reveals the Immune Responses of *Epinephelus coioides* to *sigX* Gene of *Pseudomonas plecoglossicida*

**DOI:** 10.3389/fimmu.2018.01624

**Published:** 2018-07-16

**Authors:** Yujia Sun, Gang Luo, Lingmin Zhao, Lixing Huang, Yingxue Qin, Yongquan Su, Qingpi Yan

**Affiliations:** ^1^Fisheries College, Key Laboratory of Healthy Mariculture for the East China Sea, Ministry of Agriculture, Jimei University, Xiamen, China; ^2^State Key Laboratory of Large Yellow Croaker Breeding, Ningde, China

**Keywords:** immune response, *Epinephelus coioides*, *Pseudomonas plecoglossicida*, *sigX*, RNAi, RNA-seq, miRNA, lncRNA

## Abstract

*Pseudomonas plecoglossicida* is an important pathogen for aquaculture and causes high mortality in various marine fishes. Expression of *sigX* was found significantly up-regulated at 18°C than at 28°C, which was verified by quantitative real-time PCR (qRT-PCR). RNAi significantly reduced the content of *sigX* mRNA of *P. plecoglossicida*, whether in *in vitro* or in the spleen at all sampling time points. Compared with the wild-type strain, the infection of *sigX*-RNAi strain resulted in the onset time delay, and 20% reduction in mortality of *Epinephelus coioides*, as well as alleviates in the symptoms of *E. coioides* spleen. Compared with wild-type strain, the gene silence of *sigX* in *P. plecoglossicida* resulted in a significant change in transcriptome of infected *E. coioides*. The result of gene ontology and KEGG analysis on *E. coioides* showed that genes of serine-type endopeptidase and chemokine signaling pathway, coagulation and complement system, and intestinal immune network for IgA production pathway were mostly affected by *sigX* of *P. plecoglossicida*. Meanwhile, the immune genes were associated with different number of miRNA and lncRNA, and some miRNAs were associated with more than one gene at the same time. The results indicated that *sigX* was a virulent gene of *P. plecoglossicida*. The up-regulation of the immune pathways made *E. coioides* more likely to kill *sigX*-RNAi strain than the wild-type strain of *P. plecoglossicida*, while the immune genes were regulated by miRNA and lncRNA by a complex mode.

## Introduction

*Pseudomonas plecoglossicida*, a Gram-negative aerobic rod-shaped bacteria, was first isolated and identified from cultured ayu (*Plecoglossus altivelis*) with bacterial hemorrhagic ascites (1). Recently, *P. plecoglossicida* has been associated with the fulminating infectious disease of several marine fish, such as rainbow trout (*Oncorhynchus mykiss*) and large yellow croaker (*Pseudosciaena crocea/Larimichthys crocea*) (2), and mainly recorded in the seawater temperature range from 15 to 20°C. In order to reveal the mechanism underlying the pathogenic, the transcriptome of *P. plecoglossicida* incubated under 12, 18, and 28°C were sequenced, and the data have been deposited in the NCBI Sequence Read Archive (SRA) under accession number SRP107111. The results of comparative transcriptome analysis showed that *sigX* was significantly high expressed under 18°C.

*sigX* encodes the protein SigX, a member of extracytoplasmic function sigma factors (3). SigX has been reported to play various important roles in prokaryote (4), such as iron uptake (5), fatty acid metabolism, swarming activity, adoption, c-di-GMP level maintenance, and biofilm formation (6). Deletion of *sigX* gene in *P. aeruginosa* resulted in an 18% increase in survival rate of infected *Caenorabditis elegans* compared with the wild-type strain (7). Up to now, the understanding of the function of *sigX* in bacterial pathogenicity is very limited, while no research about function of *sigX* during pathogen infection has been reported.

In view of the great harm of *P. plecoglossicida* to cultured fish and the potential important role of *sigX* in the virulence of *P. plecoglossicida*, the *sigX* of *P. plecoglossicida* was knocked down by RNAi. Then, the virulence of control and *sigX*-RNAi *P. plecoglossicida* to *Epinephelus coioides* was compared. The spleens of *E. coioides* infected by wild-type strain and *sigX*-RNAi *P. plecoglossicida* were subjected to RNA-seq, and the data were compared. The aim of this paper is to reveal the effect of *sigX* silence of *P. plecoglossicida* to the immune response of *E. coioides*.

## Materials and Methods

### Bacterial Strains and Culture Conditions

The pathogenic *P. plecoglossicida* strain (NZBD9) was isolated from the spleen of naturally infected large yellow croaker (2). The NZBD9 strain was routinely grown in LB (Luria Bertani) medium at 18 or 28°C with shaking at 220 rpm. *Escherichia coli* DH5α was obtained from TransGen Biotech (Beijing, China), which was grown in LB medium (37°C, 220 rpm).

### Construction of *P. plecoglossicida* RNAi Strain

RNAi strain was constructed according to methods described by Choi and Schweizer (8) and Darsigny et al. (9). Five short hairpin RNA sequences targeting the *sigX* gene were designed and synthesized by Shanghai Generay Biotech Co., Ltd. (Shanghai, China) (Table S1 in Supplementary Material). After linearizing pCM130/tac vectors with the restriction enzymes *Nsi*I and *Bsr*GI (New England Biolabs, USA), the oligonucleotides were annealed and ligated to the linearized pCM130/tac vectors using T4 DNA ligase (New England Biolabs) following the manufacturer’s recommendations. The recombinant pCM130/tac vectors were transformed into the competent *E. coli* DH5a cells by heat shock and then were extracted and electroporated into *P. plecoglossicida* as described previously (10). Finally, the expression level of *sigX* of each RNAi strain was detected by qRT-PCR.

### Artificial Infection and Sampling

All animal experiments were carried out strictly under the recommendations in the “Guide for the Care and Use of Laboratory Animals” set by the National Institutes of Health. The animal protocols were approved by the Animal Ethics Committee of Jimei University (Acceptance NO JMULAC201159).

Healthy *E. coioides* were obtained from Zhangzhou (Fujian, China) and were acclimatized at 18°C for 1 week under specific pathogen-free laboratory conditions. For survival assays, weight-matched *E. coioides* were intrapleurally injected with 10^3^ cfu/g of *P. plecoglossicida* (wild-type strain or RNAi strain). *E. coioides* that were intrapleurally injected with PBS were used as a negative control. The water temperature during infection was 18 ± 1°C. The daily mortality of infected *E. coioides* was recorded. For tissue RNA-Seq, the spleens of six weight-matched *E. coioides* infected with wild-type strain *P. plecoglossicida* or RNAi strain were sampled at 24 hpi (hours post infection). Every two spleens were mixed as one sample. For the tissue distribution assays, the spleens, livers, head kidneys, trunk kidneys, and blood of three *E. coioides* were sampled at 24, 48, 72, and 96 hpi, respectively.

### DNA Isolation

DNA purification from spleens, livers, head kidneys, and trunk kidneys was accomplished with an EasyPure Marine Animal Genomic DNA Kit (TransGen Biotech, Beijing, China) following the manufacturer’s instructions. The EasyPure Blood Genomic DNA Kit (TransGen Biotech) was used for DNA isolation from blood samples.

### RNA Isolation

Total RNA was extracted using TRIzol reagent (Invitrogen, Carlsbad, CA, USA) according to the manufacturer’s instructions, the mixed genomic DNA in total RNA was digested with the Turbo DNA-free DNase (Ambion, Austin, TX, USA). The RNA quality was assessed using an Agilent 2100 Bioanalyzer (Agilent Technologies, Santa Clara, CA, USA), while the rRNA in total RNA was removed using the Ribo-Zero rRNA Removal Kit (Epicentre, Madison, WI, USA) according to the manufacturer’s instructions.

### Quantitative Real-Time PCR (qRT-PCR)

Quantitative real-time PCR was carried out using a QuantStudio 6 Flex real-time PCR system (Life Technologies, USA). All primer sequences are provided in Table S2 in Supplementary Material. The expression of bacterial genes was normalized using *16s* rDNA. In *E. coioides*, the expression of mRNA and lncRNA was normalized to β*-actin*, and the miRNA expression was normalized by using *5S rRNA* gene (11). The 2^−ΔΔCt^ method was used to calculate the relative level of gene expression.

### Transcriptomic Analysis

#### Library Preparation and Sequencing

The RNA-seq libraries were prepared using protocols supplied with the TruSeq™ RNA sample preparation Kit (Illumina, San Diego, CA, USA). In brief, the rRNA-depleted RNA sample was fragmented in fragmentation buffer, and cDNA synthesis was conducted using a SuperScript double-stranded cDNA synthesis kit (Invitrogen, Carlsbad, CA, USA). After end reparation, phosphorylation, and poly (A) addition, the cDNA library was amplified using Phusion DNA polymerase (New England Biolabs). Small RNA-seq libraries were built using a TruSeq™ Small RNA Sample Prep Kit (Illumina) according to the manufacturer’s instructions. An Agilent 2100 Bioanalyzer (Agilent Technologies) was used to validate the library quality. Sequencing was performed on the Illumina HiSeq4000 sequencing platforms at Majorbio Biotech Co., Ltd. (Shanghai, China).

#### Processing and Mapping of Reads

The trimming and quality control of raw Illumina reads were performed using Sickle^1^ and SeqPrep^2^ with the default settings. For RNA-seq, clean data were mapped to the genome of *P. plecoglossicida* strain NZBD9 (NCBI SRA under accession number SRP062985) using Bowtie2 (12). Mapped reads were classified as reads of *P. plecoglossicida*, and leftover reads were used for *de novo* assembly to obtain the *E. coioides* unigenes.

#### *De Novo* Assembly, Detection of lncRNAs, and Annotation of mRNAs in the Host

All clean reads, which were not mapped to the *P. plecoglossicida* genome from the wild-type strain and RNAi strain-infected spleens, were treated as a pool of reads. This pool of reads was assembled *de novo* into unigenes using Trinity (13). To remove any possible prokaryote contamination, all unigenes were first aligned to the bacterial NCBI non-redundant (NR) protein database. Next, the detection of lncRNAs was performed as described earlier (14). For annotation of mRNAs, the clean unigenes were compared against different databases, including NCBI NR protein, STRING, SWISS-PROT, and KEGG (Kyoto Encyclopedia of Genes and Genomes) databases using BLASTX to identify the proteins that shared the greatest sequence similarity with the identified unigenes. Gene ontology (GO) annotations were conducted using the Blast2GO software (15).^3^ Finally, KEGG was used for metabolic pathway analysis (16).^4^

#### Identification of miRNAs

The raw reads were processed through quality control to obtain clean reads. The clean reads were then blasted against the Rfam database^5^ to annotate the miscellaneous RNAs. After filtering rRNA, snoRNA, scRNA, tRNA, snRNA, and other non-coding RNA, the remaining sRNAs were mapped to the zebrafish miRNA data of miRBase version 21.0 to detect the known and new miRNAs.

#### Analysis of Differential Gene Expression

Expression analyses of transcriptome data from *E. coioides* were performed on the basis of annotations from NCBI (NZ_ASJX00000000.1) and the reference transcriptome annotation described above (annotation for the host transcriptome), respectively. After obtaining uniquely mapped read counts, the package edgeR (targeting mRNAs of host and lncRNAs of host) (17) and DESeq2 (targeting miRNAs of host) (18) were used to test for differentially expressed genes (DEGs), which were determined by the following thresholds: |log2^fold change^| ≥1 and a false discovery rate (FDR) <0.05.

### Statistical Analyses

All data are expressed as the mean ± SD from at least three sets of independent experiments. Data analysis was performed using the SPSS 17.0 software (Chicago, IL, USA), and one-way analysis of variance with Dunnett’s test was used. *P* values <0.05 were considered statistically significant.

### Data Access

The RNA sequencing reads data were deposited at the GenBank SRA database under the accession numbers SRP140359 and SRP115064.

## Results

### Construction of the *sigX*-RNAi Strain

**Figure 1:**
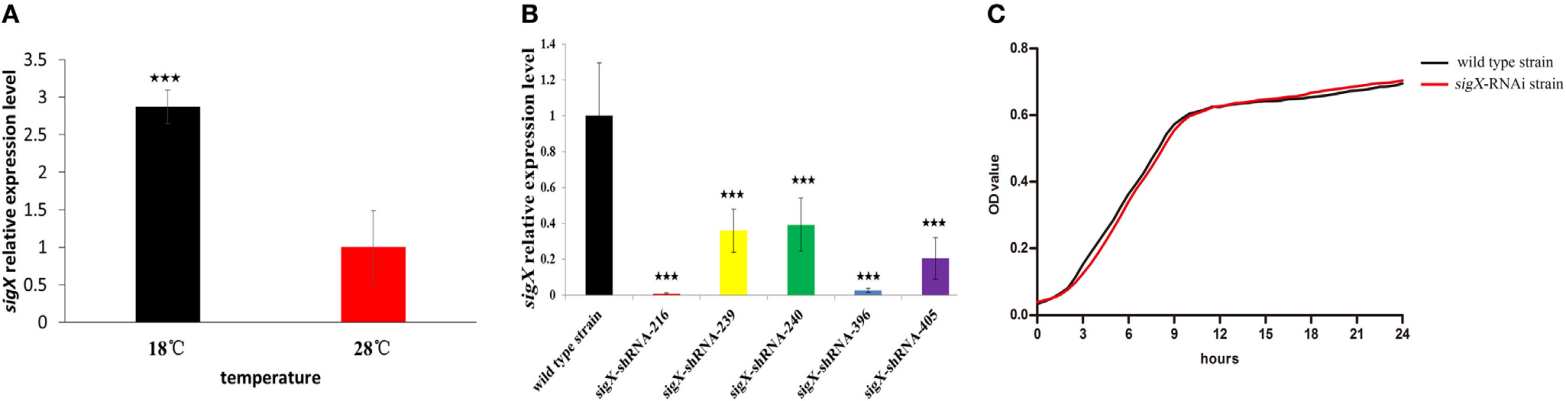
Construction and growth curve of *sigX*-RNAi strain of *Pseudomonas plecoglossicida*. **(A)** Expression of *sigX* mRNA at 28 and 18°C in wild-type strain. **(B)** The *sigX* mRNA levels of 5 *sigX*-RNAi silencing strain. **(C)** Growth curve of wild-type strain and *sigX*-RNAi strain. ^***^P ≤ 0.001.

Figure 1A exhibits the qRT-PCR results of *sigX* gene expression of *P. plecoglossicida* cultured *in vitro* under different temperature. The results showed that the expression of *sigX* gene was significantly higher at 18^°^C than at 28^°^C (Figure 1A), which was consistent with the results of previous RNA-seq (19).

All of the five shRNAs targeted on sequences of *sigX* gene are shown in Figure S1 in Supplementary Material, and all of them extremely significantly reduced the mRNA expression of *sigX* gene, with different efficiency (Figure 1B). The strain containing pCM130/tac-*sigX*-shRNA-216 (named the *sigX*-RNAi strain) exhibited the best efficiency of gene silencing, and was chosen for further studies. Despite *sigX* has been silenced, *sigX*-RNAi strain growth rate is almost the same as control strain (Figure 1C).

### The Effect of *sigX* Gene on the Virulence of *P. plecoglossicida*

**Figure 2:**
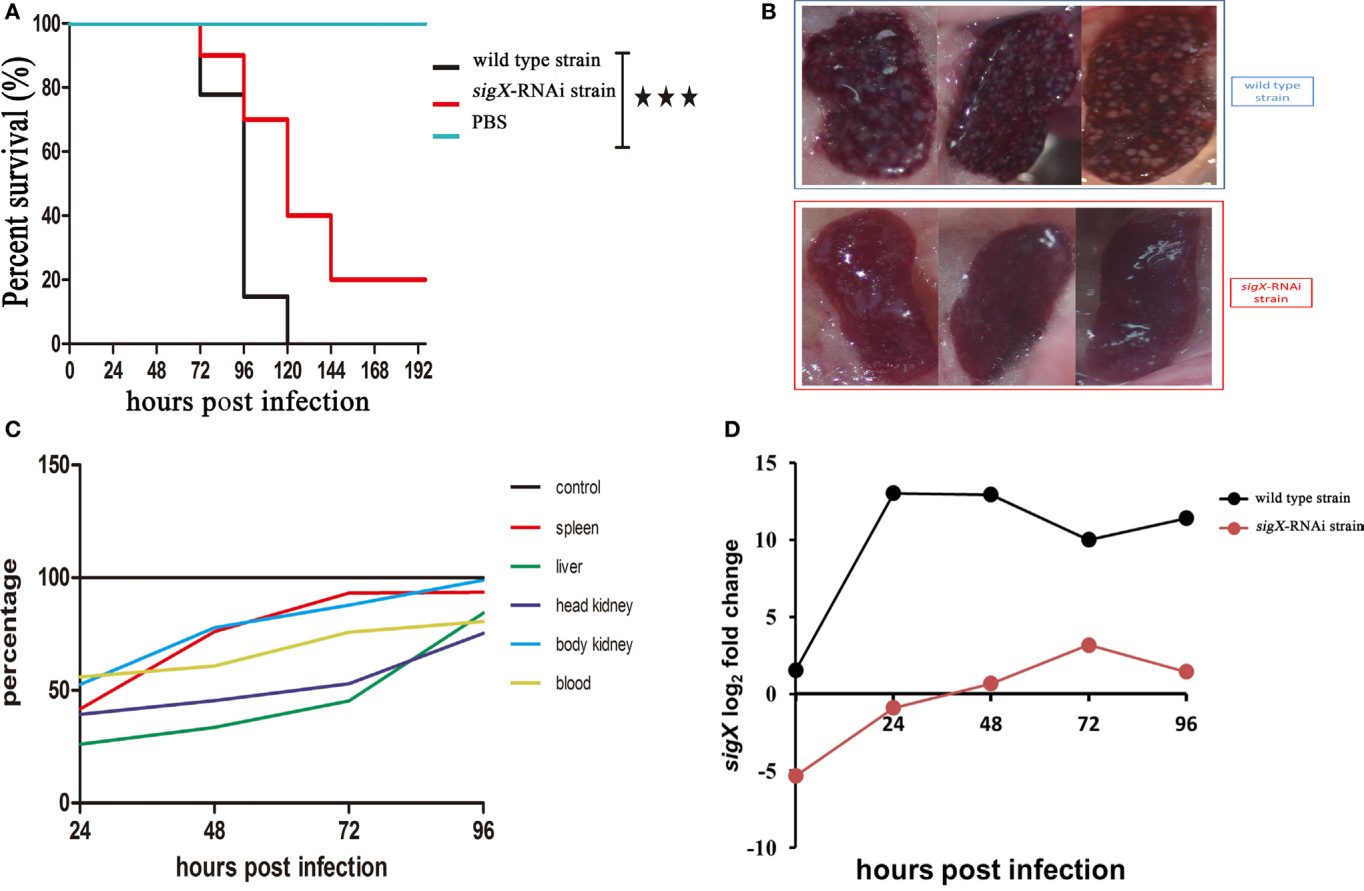
The virulence of wild-type strain and *sigX*-RNAi strain of *Pseudomonas plecoglossicida*. **(A)** Survival rate of *Epinephelus coioides* infected by *P. plecoglossicida*. **(B)** Symptoms of spleen of *E. coioides* infected by *P. plecoglossicida*. **(C)** Spatial and temporal distribution of *sigX*-RNAi strain *P. plecoglossicida* compared to wild-type strain. **(D)** Expression level of *sigX* gene of *P. plecoglossicida* in the spleen of *E. coioides*.

Compared with the counterparts injected with wild-type strain of *P. plecoglossicida*, the *E. coioides* injected with *sigX*-RNAi strain exhibited a significantly delay in the time of death and a significantly decrease in mortality (Figure 2A). At 96 hpi, the spleens of the *E. coioides* injected with wild-type strain of *P. plecoglossicida* appeared typical symptoms (the surface of the spleen is covered with numerous white spots), however, some less obvious white spots were found on the surface of spleens of *E. coioides* injected with RNAi strain (Figure 2B).

Figure 2C shows the difference in dynamic distribution of *P. plecoglossicida* between *E. coioides* infected with *sigX*-RNAi strain and wild-type strain of *P. plecoglossicida*. The percentage of *sigX*-RNAi strain abundance to wild-type strain abundance varies according to organ and time, and increased with the time post infection. The percentage of *sigX*-RNAi strain abundance to wild-type strain abundance in the spleen and body kidney was much higher than those in the head kidney and liver. At 96 hpi, the abundance of *sigX*-RNAi strain in the spleen and body kidney was close to those of wild-type strain.

Figure 2D shows the expression level of *sigX* gene of *sigX*-RNAi strain and wild-type strain of *P. plecoglossicida in vitro* and spleens at different times post-injection under 18^°^C. The expression level of *sigX* gene in the spleen infected with wild-type strain increased to a peak at 24 hpi and then decreased gradually. However, when infected with *sigX*-RNAi strain, the expression increased gradually until 72 hpi. The expression of both strains was higher *in vivo* than *in vitro*. The expression of *sigX* in spleen infected with *sigX*-RNAi strain at 72 hpi was slightly higher than wild-type strain *in vitro*.

### Tissue RNA-Seq of Infected Spleen of *E. coioides*

#### Differentially Expressed Genes

**Figure 3:**
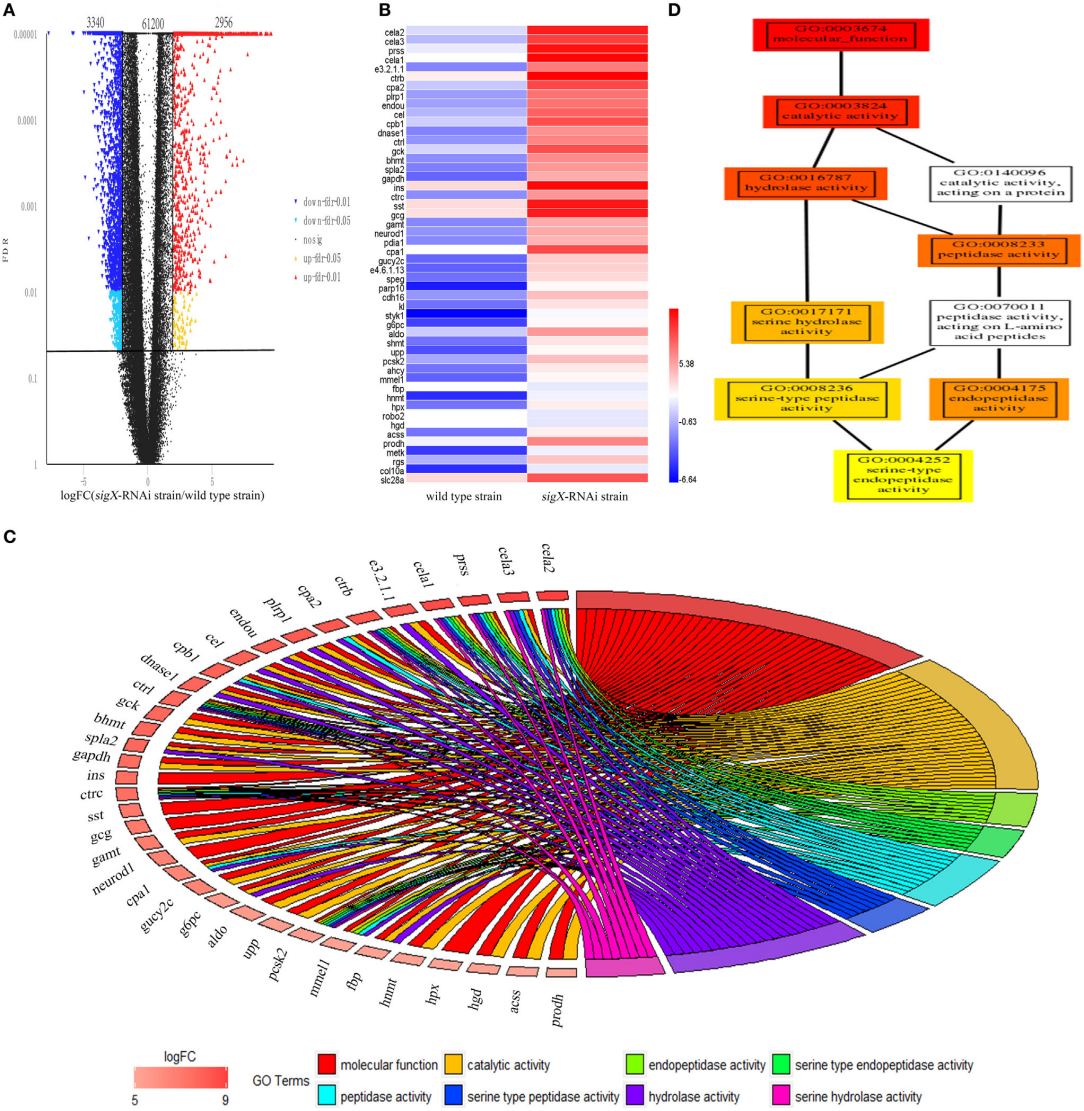
Differentially expressed genes (DEGs) of *Epinephelus pinephelus* infected by *Pseudomonas plecoglossicida* and gene ontology (GO) analysis. **(A)** Volcano plot of all genes, *X*-axis represent the fold change values of samples infected by *sigX*-RNAi strain/samples infected by wild-type strain, *Y*-axis represent statistical test value [false discovery rate (FDR)], the higher represent the more significant differences. Each dot represents a particular gene: the red dots indicate significantly up-regulated genes; the blue dots represent significant down-regulated genes; and the black dots represent non-significant differences genes. **(B)** Heat map of the top 50 up-regulated genes (adjusted FDR < 0.05; ǀ log2FC ǀ ≥ 1; 3 biological replicates) between wild-type strain and *sigX*-RNAi strain infections. Values represent log2 folds. Colors based on log-transformed transcripts FPKM mean values. Blue and red indicate decreased and increased expression, respectively. **(C)** Chordal graph of top 50 DEGs to GO terms. **(D)** GO-directed acyclic graph chart of 8 GO terms which belongs to molecular function. Each node at the right half of the circle represents one of the 8 GO term, each node at the left half of the circle represents a gene. The degree of colors represents the enrichment significance levels according to *P*-value. The redder the color of the gene, the greater the up-regulation of the expression.

High-quality reads is the foundation of RNA-Seq approach. The base distribution is balance, and N% is within the reasonable range (Figure S2 in Supplementary Material). The reproducibility of the three biological duplicates is satisfactory (Figure S3 in Supplementary Material). The gene expression profile was calculated by edgeR, and the changes in the expression level met FDR < 0.05 and |log2FC| ≥1 were considered statistically significant difference. 67,496 mRNAs were identified from profiled transcripts of the spleen infected by the *sigX*-RNAi strain. Compared with the spleen infected by the wild-type strain, 6,296 mRNAs from the spleen infected by the *sigX*-RNAi strain exhibited significant difference in abundance with 3,340 mRNAs down-regulated and 2,956 mRNAs up-regulated (Figure 3A). According to logFC, the top 50 up-regulated DEGs were picked out and shown in Figure 3B. Among the top 50 up-regulated DEGs, 17 genes could not match to GO terms, and 33 genes matched to GO terms successfully, while the top 8 GO terms belongs to are molecular function, catalytic activity, endopeptidase activity, serine-type endopeptidase activity, peptidase activity, serine-type peptidase activity, hydrolase activity, and serine hydrolase activity, respectively. Relationships between genes and GO terms are shown in Figure 3C. GO-directed acyclic graph was used to analyze the relationship among these 8 terms, which showed that serine-type endopeptidase activity was the most detailed term (Figure 3D). Serine-type endopeptidase activity enriched 7 up-regulated genes, they are chymotrypsin-like elastase family, member 1 (*cela1*), chymotrypsin-like elastase family, member 2 (*cela2*), chymotrypsinogen 2, partial (*ctrb*), chymotrypsin C (*ctrc*), chymotrypsinogen (*ctrl*), trypsinogen (*prss*), and neuroendocrine convertase 2 (*pcsk2*).

**Figure 4:**
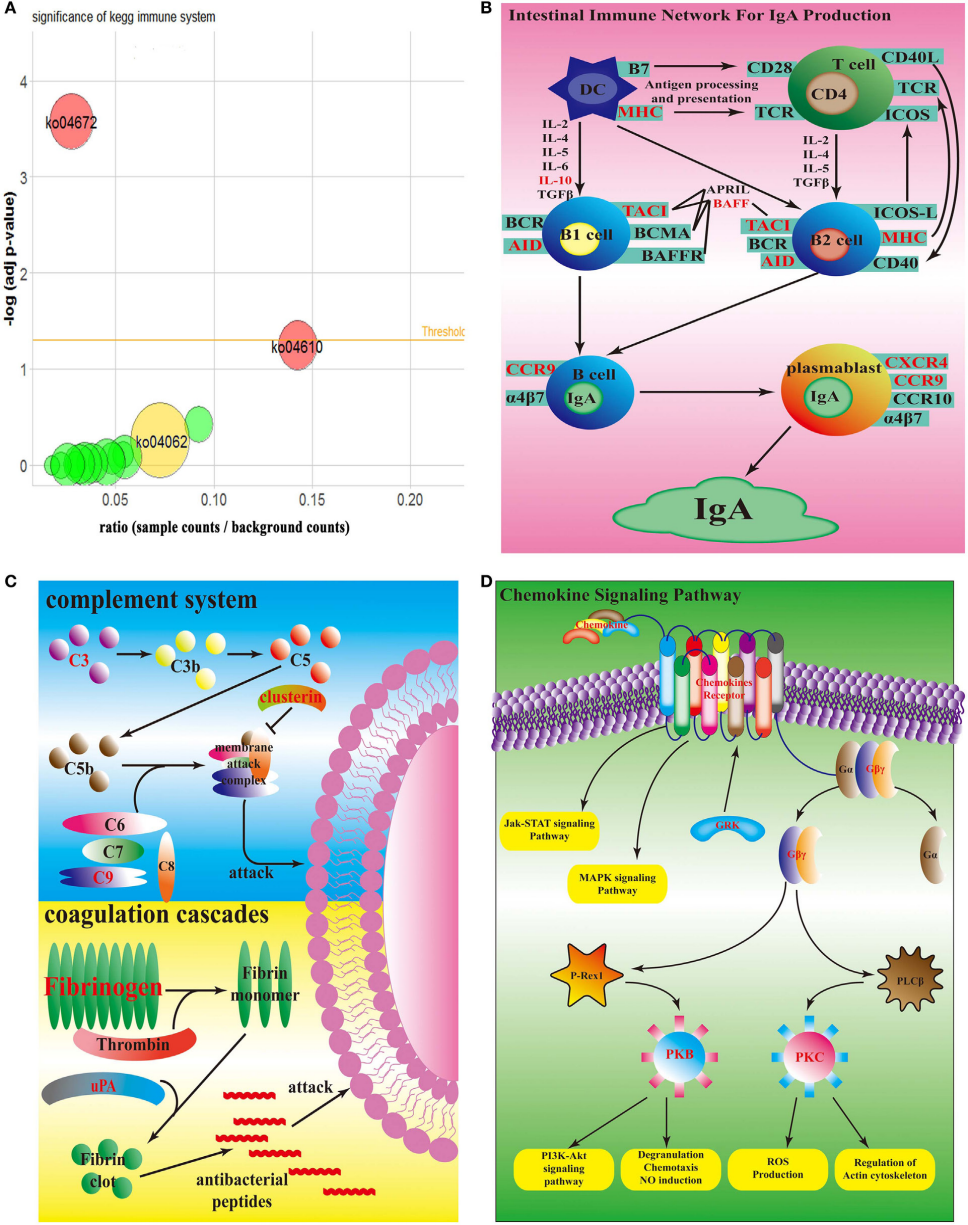
Top enrichment KEGG pathway. **(A)** Bubble graph of top enrichment KEGG pathway. Red bubbles are remarkable pathway (adj *p*-value <0.05); yellow bubble is the pathway which has the most number of differentially expressed genes; green bubbles are other pathways. **(B)** Simplified overview of *sigX*-RNAi strain interacting with the host intestinal immune network for IgA pathway. **(C)** Simplified overview of *sigX*-RNAi strain interacting with the host coagulation and complement system. **(D)** Simplified overview of *sigX*-RNAi strain interacting with the host chemokine signaling pathway. The black letter genes in **(B–D)** represent no different expression and the red letter genes in **(B–D)** are up-regulated in spleen infected by *sigX*-RNAi strain compared with the counterpart infected by wild-type strain.

According to KEGG database, all 50 top up-regulated DEGs were enriched in 15 KEGG pathways, which were all relative to immune system. Compared with the counterpart in the spleen infected by wild-type strain, all of these 15 pathways were up-regulated in spleen infected by *sigX*-RNAi strain (Figure 4A). According to *P*-value of Fisher’s exact test, ko04672, which represents intestinal immune network for IgA production, was significant. ko04610, a pathway of coagulation and complement system, had the highest ratio. ko04062, the chemokine signaling pathway, enriched the largest number of DEGs. In intestinal immune network for IgA production pathway, genes, MHC class II antigen beta chain (*mhc2*), TNF receptor superfamily member 13B (*tnfrsf13b*), activation-induced cytidine deaminase (*aicda*), C-C chemokine receptor type 9 (*ccr9*), C-X-C chemokine receptor type 4 (*cxcr4*), and interleukin-10 (*il10*), which encode MHC, TACI, AID, CCR9, CXCR4, and IL10, respectively, were up-regulated (Figure 4B). In coagulation and complement system, genes, complement component c3 (*c3*), complement component c9 (*c9*), clusterin *(clu)*, fibrinogen alpha chain (*fga*), fibrinogen beta chain (*fgb*), fibrinogen gamma chain (*fgg*), urokinase-type plasminogen activator (*plau*), which encode C3, C9, clusterin, fibrinogen, and uPA, were up-regulated (Figure 4C). In chemokine signaling pathway, genes, C-C motif chemokine 4 (*ccl4*), C-C motif chemokine 7 (*ccl7*), C-C motif chemokine 19 (*ccl19*), C-C motif chemokine 20 (*ccl20*), C-X-C motif chemokine 1 (*cxcl1*), C-X-C motif chemokine 8 (*cxcl8*), C-X-C chemokine receptor type 2 (*cxcr2*), C-X-C chemokine receptor type 3 (*cxcr3*), *cxcr4*, chemokine XC receptor 1 (*xcr1*), *ccr9*, guanine nucleotide-binding protein (G protein), gamma 2 (*gng2*), guanine nucleotide-binding protein subunit beta-5 (*gnb5*), rhodopsin kinase 1 (*grk1*), Rac protein (*rac2*), and protein kinase C delta type (*prkcd*), which encode chemokines, chemokine receptors, Gβγ, GRK, PKB, and PKC, were up-regulated (Figure 4D). There were no down-regulated genes enriched in these three pathways.

#### Non-Coding RNA

lncRNAs were authenticated by coding potential calculator and coding-non-coding index, and the collection was taken for further research. A total of 38,813 lncRNA were authenticated from the spleen infected by the *sigX*-RNAi strain (Figure S4A in Supplementary Material). Compared with the spleen infected by the wild-type strain, 8,668 lncRNA from the spleen infected by the *sigX*-RNAi strain were down-regulated, and 4,788 lncRNA were up-regulated, while the other 25,357 lncRNA exhibited no significant change (Figure 4B in Supplementary Material).

miRNAs were authenticated by Rfam database. A total of 562 miRNA were authenticated from the spleen infected by the *sigX*-RNAi strain. Compared with the spleen infected by the wild-type strain, only 58 miRNAs from the spleen infected by the *sigX*-RNAi strain exhibited significant change in expression quantity. Among these 58 differently expressed miRNAs, 10 were known, and the other 48 were novel miRNA. Among them, 39 novel miRNAs and 4 known miRNAs were down-regulated, while 9 novel miRNAs and 6 known miRNAs were up-regulated (Figures S5A,B in Supplementary Material).

Some transcripts were selected from transcriptome and confirmed by qRT-PCR, including mRNA, lncRNA, and miRNA (Figures S6A–C in Supplementary Material).

#### Network of Differentially Expressed miRNA–mRNA–lncRNA Relationship

**Figure 5:**
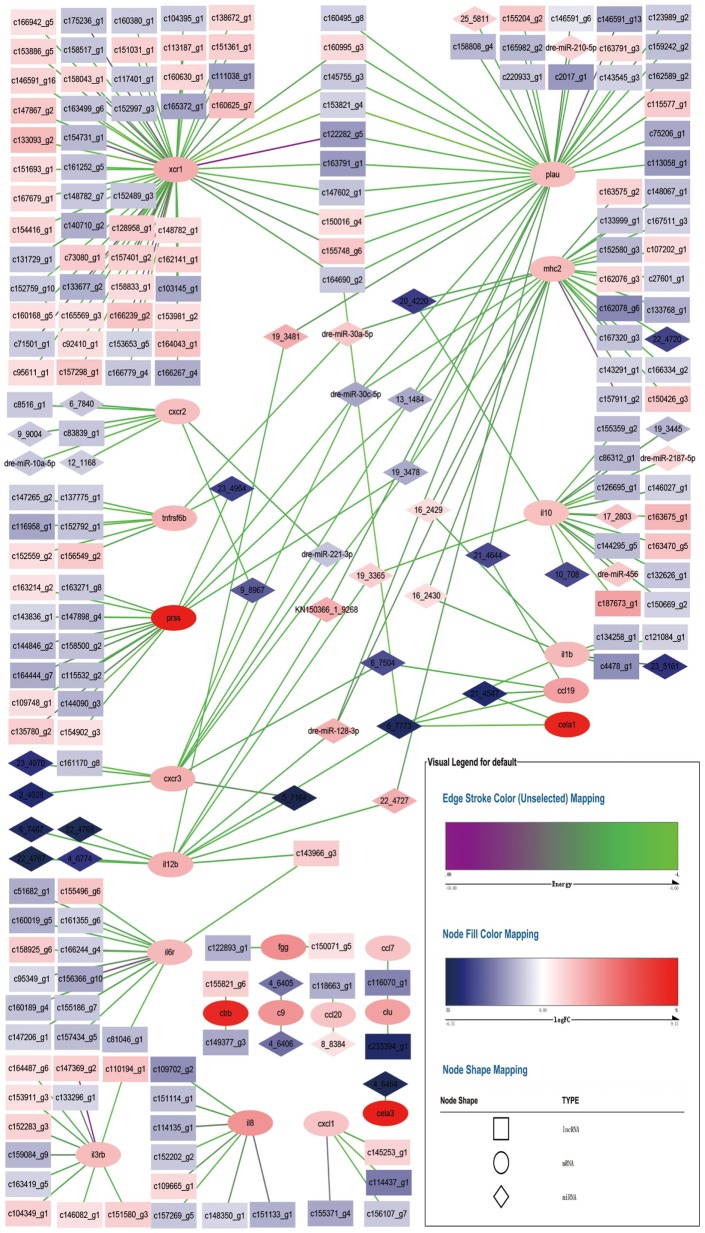
Network for differentially expressed miRNA–mRNA–lncRNA relationship. Square nodes represent differentially expressed lncRNAs, circle nodes represent differentially expressed mRNAs, and rhombus nodes represent differentially expressed miRNAs. Red color of nodes represent up-regulate and blue color of nodes represent down-regulate. The darker the color, the greater the change. Edges color range from purple to green represent interact energy from low to high. The smaller the purple energy, the closer the bond.

Figure 5 shows the relationships of 23 DEGs with differentially expressed miRNA and differentially expressed lncRNA. Each DEG was associated with different number of differentially expressed non-coding RNA. For example, *xcr1* was the target of 60 lncRNA, while *cxcr2* was the target of 2 lncRNA and 6 miRNA.

Each DEG was associated with at least one other DEG through non-coding RNA, except *fgg, ctrb, c9, ccl20, cela3, cxcl1, il8*, and *il3rb*. *xcr1* and *plau* shared 9 lncRNA, lncRNA c164690_g2 also tied to *cela1, il1b, il12b*, and *ccl19 via* a miRNA. *plau, mhc2, prss*, and *cxcr3* shared 3 miRNA. *plau* and *il12b* shared 2 miRNA. *plau, mhc2*, and *il12b* shared 1 miRNA. *mhc2* and *il10* shared 1 miRNA. *mhc2* and *tnfrsf6b* shared 1 miRNA. *mhc2* and *il1b* shared 2 miRNA. *mhc2* and *ccl19* shared 1 miRNA. *il10, il12b*, and *cela1* shared 1 miRNA. *ccl19* and *cxcr3* shared 1 miRNA. *ccl19* and *cela1* shared 1 miRNA. *il12b* and *il6r* shared 1 lncRNA. *il12b* and *cxcr3* shared 1 miRNA. *cxcr2* and *cxcr3* shared 2 miRNA. *prss* and *tnfrsf6b* shared 1 miRNA. *clu* and *ccl7* shared 1 lncRNA. Compared with the counterpart in *E. coioides* spleen infected by the wild-type strain of *P. plecoglossicida*, all of the non-coding RNA had different expression levels in *E. coioides* spleen infected by the *sigX*-RNAi strain. Although all of the immune DEGs were up-regulated in *E. coioides* spleen infected by the *sigX*-RNAi strain of *P. plecoglossicida*, compared with the counterpart in *E. coioides* spleen infected by the wild-type strain, only 102 out of 278 non-coding RNA were up-regulated.

## Discussion

Most pathogens cause disease by toxicity and invasiveness, which are regulated by different genes (20). So far, a lot of genes have been verified to be involved in the virulence regulation of aquatic pathogenic bacteria by knockout or knockdown technology, such as *flgE* to *Aeromonas hydrophila* (21); *flrA flrB* and *flrC* to *Vibrio alginolyticus* (10); s*ecA secD secF yajC* and *yidC* to *Vibrio alginolyticus* (22); and *oppABCDF* to *Vibrio alginolyticus* (23). However, no studies on contribution of *P. plecoglossicida sigX* gene to virulence have been reported.

RNAi is a highly efficient and specific tool to research gene function (24). In the present study, 5 shRNA were designed for 5 different targets of *sigX*. All of the 5 shRNA significantly reduced the content of *sigX* mRNA and the shRNA for target started at 216 exhibited the best efficiency of gene silencing with a reduction of 99.27% (Figure 1B). The content of *sigX* mRNA of *P. plecoglossicida* in spleen infected by *sigX*-RNAi strain was less than 1% of the counterpart in spleen infected by wild-type strain at all sampling times (Figure 2D), which indicated the high stability of *sigX* silence throughout the infection process. Therefore, the stable silencing technology in the present study laid the foundation for the follow-up of *sigX* gene function.

The pathogenicity of *P. plecoglossicida* was significantly temperature-dependent, while water temperature of 15–20°C was the high pathogenic temperature (2). The expression of *sigX* in *P. plecoglossicida* was significantly higher at 18°C than at a rapid growth temperature (28°C) (Figure 1A), which indicated that *sigX* might be related to the virulence of *P. plecoglossicida*. Compared with the wild-type strain, the infection of *sigX*-RNAi strain resulted in the onset time delay, and 20% reduction in mortality of *E. coioides* (Figure 2A), as well as alleviates in the symptoms of *E. coioides* spleen (Figure 2B). The results suggested that gene silencing of *sigX* led to the decrease in virulence of *P. plecoglossicida*. Spleen was the main target organ of *P. plecoglossicida* (2), the symptoms of *E. coioides* spleen caused by *P. plecoglossicida* infection were significantly different between the wild-type strain and *sigX*-RNAi strain (Figure 2C), which suggest spleen is a good material for studying the response of *E. coioides* immune system to bacterial infection.

It is well known that the infection of pathogen can cause great changes in the host transcriptome (25). Recently, it has been found that the change of single virulence gene can also cause significant changes in host transcriptome (26). In the present study, compared with wild-type strain, the gene silence of *sigX* in *P. plecoglossicida* resulted in a significant change in transcriptome of infected *E. coioides*. The results indicated that *sigX* of *P. plecoglossicida* had important effect on the transcriptome of *E. coioides*. The result of GO and KEGG analysis showed that serine-type endopeptidase activity GO term (*cela1, cela2, ctrb, ctrc, ctrl, prss*, and *pcsk2*) and chemokine signaling pathway (*ccl4, ccl7, ccl19, ccl20, cxcl1, cxcl8, cxcr2, cxcr3, cxcr4, xcr1, ccr9, gng2, gnb5, grk1, rac2*, and *prkcd*), coagulation and complement system (*c3, c9, clu, fga, fgb, fgg*, and *plau*), and intestinal immune network for IgA production pathway (*mhc2, tnfrsf13b, aicda, ccr9, cxcr4*, and *il10*) were most affected by *sigX* of *P. plecoglossicida*. Serine protease in neutrophils is critical for protecting host against bacterial infection (27). Serine protease was proved to have direct killing effect on *Klebsiella pneumoniae* and *E. coli* (28, 29); the presence of serine protease inhibitors inhibited the cytotoxicity of neutrophils to *Streptococcus pneumonia* (30). Chemokines cooperated with their receptors controlled the migration and residence of all immune cells, and they tend to be up-regulated usually in case of bacterial infection, and cause inflammation (31). *Mycoplasma hyopneumoniae* infection resulted in a significant up-regulation of chemokine signaling pathway in porcine alveolar macrophage. Coagulation cascades and complement systems are two distinct but cross-regulated innate immune pathways to protect host from pathogen invasion (32). In the process of killing bacteria, complement assemble membrane attack complex to react on bacterial surface; coagulation factors pick bacteria into the clots; and then generate small antibacterial peptides (33). If a pathogen is combined with immunoglobulin or complement, it is significantly more likely to be identified and phagocytosed by innate immune cells (34). *A. hydrophila* infection resulted in the up-regulation of pathway of coagulation and complement system in *Ctenopharyngodon idella* (35); *Cryptocaryon irritans* infection resulted in the up-regulation of the pathway of coagulation and complement system and chemokine signaling pathway in *Sebastiscus marmoratus* (36). The IgA generated in intestinal network is the first line to defense bacteria invasion (37). Although there have been reports about the response of these pathways to bacterial infection, no report has been found about the response of these pathways to a single gene during bacterial infection. The up-regulation of these pathways in spleen infected by *sigX*-RNAi strain indicated that *E. coioides* was more likely to kill *sigX*-RNAi strain than the wild-type strain of *P. plecoglossicida*. These results also partly explained why the *sigX*-RNAi strain was less virulent and less abundant in *E. coioides*.

The relation between mRNA and non-coding RNA is complex (38). miRNAs had been proved to play essential roles in immune response (39). It is speculated that miRNA acted as a trigger, because the characteristic of miRNA is short and miRNA can act on multiple mRNAs at the same time (39). The function and mechanism of lncRNA is complex (40). It was speculated that lncRNA had a competitive relationship with miRNA when interacting with mRNA (40). In the represent study, the immune genes were associated with different number of miRNA and lncRNA, and some miRNA associated with more than one gene at the same time. The results indicated that the immune genes were regulated by miRNA and lncRNA by a complex mode.

## Conclusion

*sigX* was a virulent gene of *P. plecoglossicida*, and contributed greatly to the virulence of *P. plecoglossicida* on *E. coioides*. Compared with the infection of wild-type strain, infection of *E. coioides* with *sigX*-RNAi strain resulted in up-regulation of genes in chemokine signaling pathway, coagulation and complement system, intestinal immune network for IgA production pathway, and as well as serine-type endopeptidase in spleen. The immune genes were regulated by miRNA and lncRNA by a complex mode.

## Author Contributions

Seven authors contributed to the article, QY and YS conceived the experiments. YS, GL, LZ, and LH conducted the experiments. All authors assisted in the collection and interpretation of data. YS and QY wrote the manuscript.

## Conflict of Interest Statement

The authors declare that the research was conducted in the absence of any commercial or financial relationships that could be construed as a potential conflict of interest.

## Footnotes

^1^https://github.com/najoshi/sickle.

^2^https://github.com/jstjohn/SeqPrep.

^3^http://www.blast2go.com/b2ghome.

^4^http://www.genome.jp/kegg/.

^5^http://Rfam.sanger.ac.uk/.
