# Supplementary material for: Integration of RNAi and RNA-seq Reveals the Immune Responses of Epinephelus coioides to sigX Gene of Pseudomonas plecoglossicida
Source: Front Immunol. 2018 Jul 16;9:1624. doi: 10.3389/fimmu.2018.01624 (PMC6054955; doi:10.3389/fimmu.2018.01624)
Supplement: Supplementary file 1 [file Image_1.PDF]

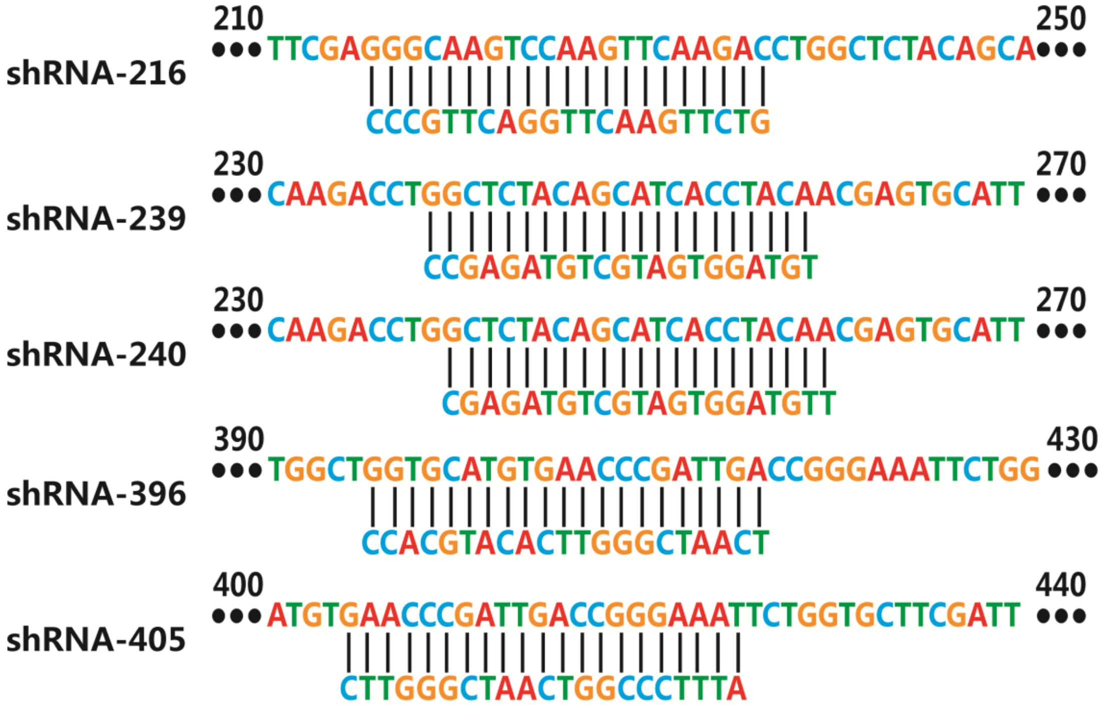

Supplementary Figure 1 shRNA targeted sequences on *sigX* gene

5 shRNA were choosen to target *sigX*. They start at 216, 239, 240, 396, 400 base of *sigX* sequence respectively.
