# Supplementary material for: Integration of RNAi and RNA-seq Reveals the Immune Responses of Epinephelus coioides to sigX Gene of Pseudomonas plecoglossicida
Source: Front Immunol. 2018 Jul 16;9:1624. doi: 10.3389/fimmu.2018.01624 (PMC6054955; doi:10.3389/fimmu.2018.01624)
Supplement: Supplementary file 2 [file Image_2.PDF]

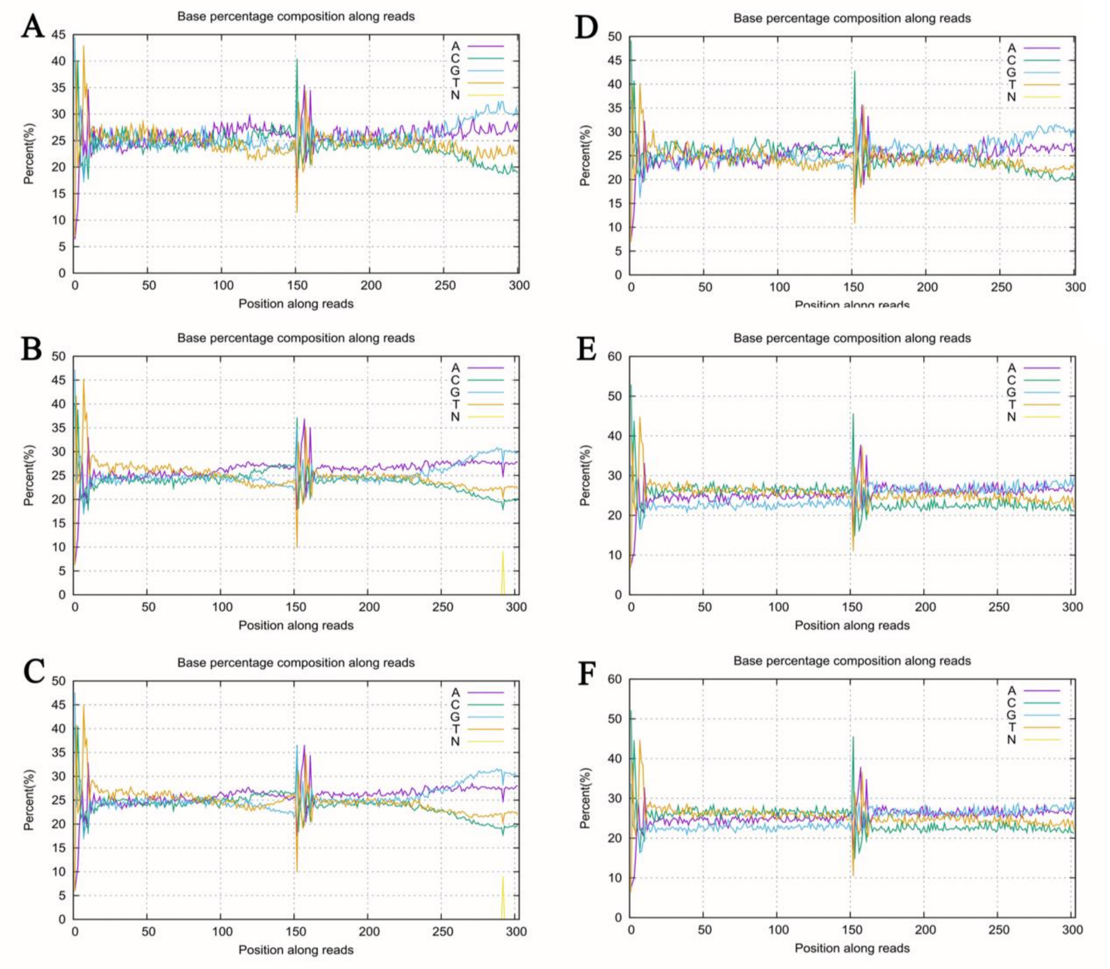

Supplementary Figure 2 Base percentage composition along reads

Horizontal axis is base coordinates of reads, which represent the base from the 5' to the 3' end sequentially. Vertical axis is corresponding percentage, each base with different color. A is purple; C is green; G is blue; T is orange; N is yellow. But in existing high-throughput sequencing technologies, reverse transcribed into cDNA was used in 6 bp random primers can cause a few position of nucleotide composition exist certain preferences, which belongs to the normal situation. **(A)**, **(B)**, **(C)** are wild type strain; **(D)**, **(E)**, **(F)** are *sigX*-RNAi strain.
