# Supplementary material for: Integration of RNAi and RNA-seq Reveals the Immune Responses of Epinephelus coioides to sigX Gene of Pseudomonas plecoglossicida
Source: Front Immunol. 2018 Jul 16;9:1624. doi: 10.3389/fimmu.2018.01624 (PMC6054955; doi:10.3389/fimmu.2018.01624)
Supplement: Supplementary file 3 [file Image_3.PDF]

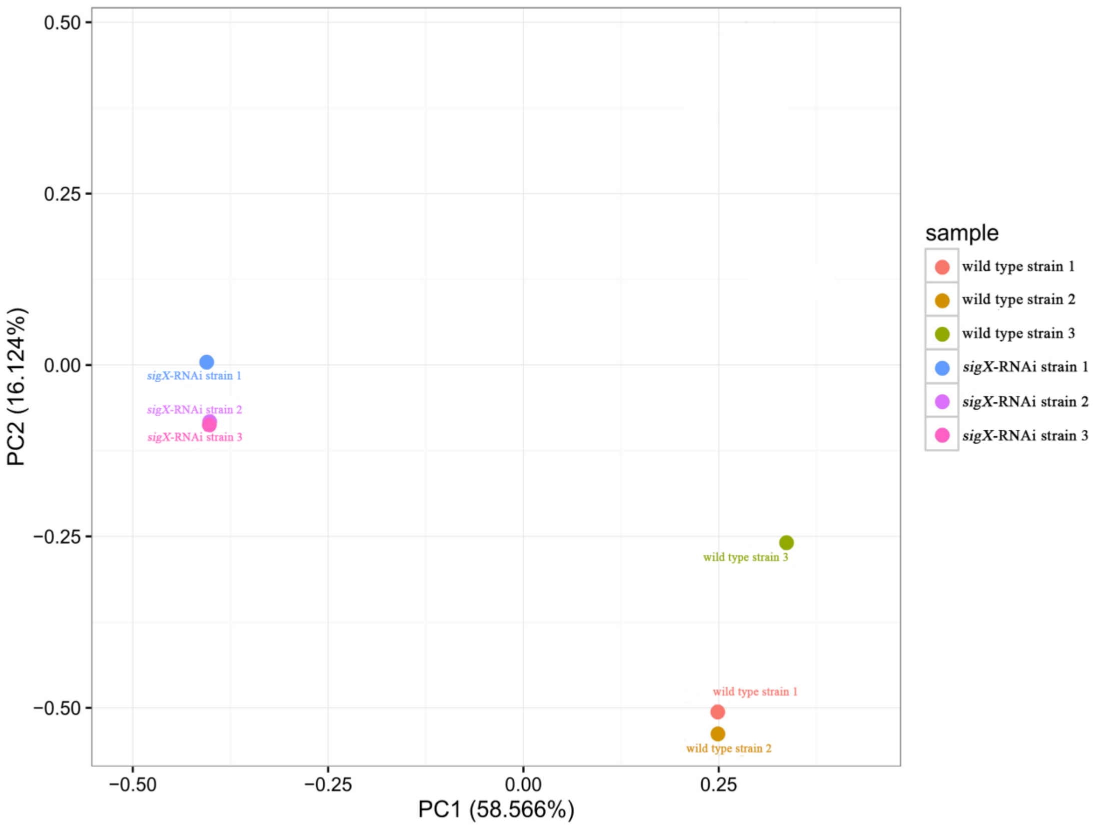

Supplementary Figure 3 PCA analysis

Principal component analysis(PCA) can reduce the complexity of data, and dig deep the relation between sample size and variation. The basic principle is that diverse samples have different measurement, PCA is to find out the main factors of observed value differences, considering all the factors are combined and sort according to importance. Usually the tiny factors are ignored, which play a role of simplify the data. For two or three principal components axis graphed, which can see the distance of the relationship between each sample, including visual effect of clusters groups.
