# Supplementary material for: Integration of RNAi and RNA-seq Reveals the Immune Responses of Epinephelus coioides to sigX Gene of Pseudomonas plecoglossicida
Source: Front Immunol. 2018 Jul 16;9:1624. doi: 10.3389/fimmu.2018.01624 (PMC6054955; doi:10.3389/fimmu.2018.01624)
Supplement: Supplementary file 4 [file Image_4.PDF]

A

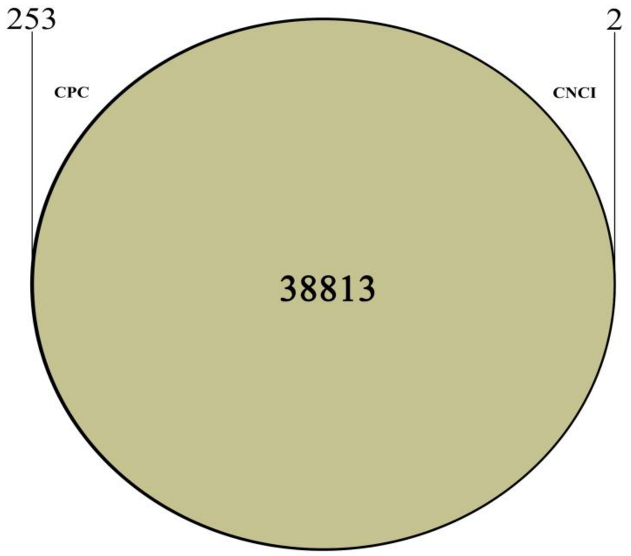

B

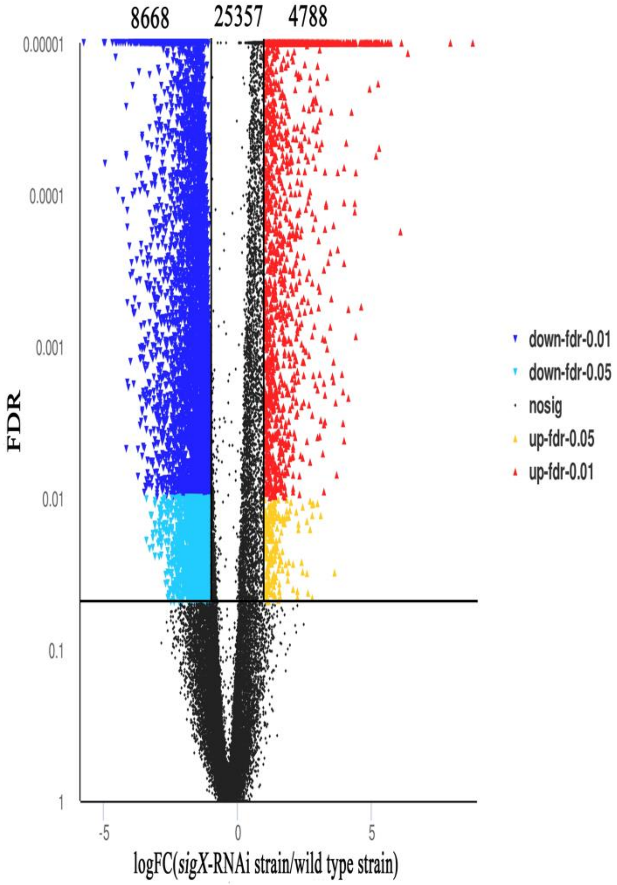

Supplementary Figure 4 lncRNA authentication and different expression lncRNA

(A): venn display of lncRNA authentication result, the circles with 2 colors represent 2 methods. CPC (Coding Potential Calculator) analysis is a protein Coding Potential computing tool based on sequence alignment. CNCI (Coding- non-coding Index) analysis is a method to distinguish Coding- non-coding transcripts by the characteristics of adjacent nucleotide triplets. The circle's cross area represents the

number of lncRNA in the two methods, meanwhile CPC score<0 as well as CNCI score<0; **(B)**: volcano plot, X-axis represent the fold change values between the two samples, the expression of wild type sample divided by the processing sample, Y-axis represent statistical test value (FDR), the higher represent the more significant differences. Two coordinate values are subjected to log treatment. Each dot represents a particular lncRNA, the red dot indicates significantly up-regulated lncRNAs, the blue dot represents a significant down-regulated lncRNAs, the black dots represent non-significant differences lncRNAs. The dot in the left represent down-regulated lncRNAs, the dot in the right represent up-regulated lncRNAs
