# Supplementary material for: Integration of RNAi and RNA-seq Reveals the Immune Responses of Epinephelus coioides to sigX Gene of Pseudomonas plecoglossicida
Source: Front Immunol. 2018 Jul 16;9:1624. doi: 10.3389/fimmu.2018.01624 (PMC6054955; doi:10.3389/fimmu.2018.01624)
Supplement: Supplementary file 5 [file Image_5.PDF]

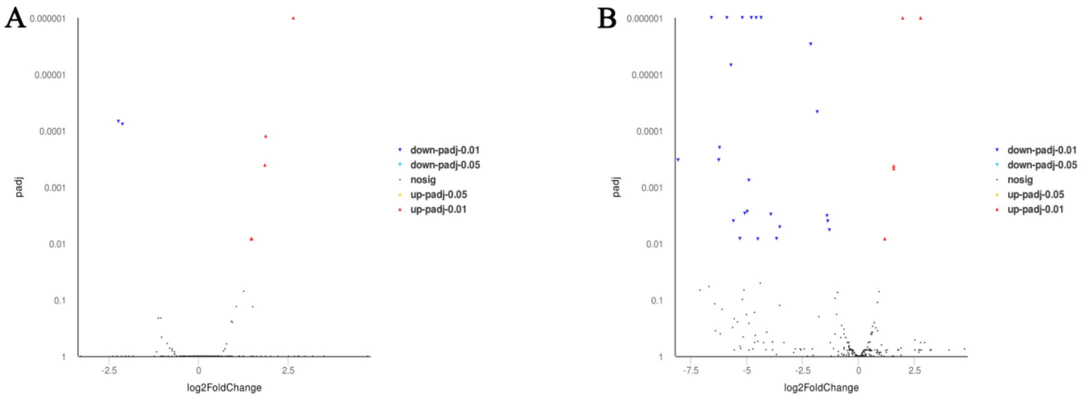

Supplementary Figure 5 miRNA volcano plot

Volcano plot of (A): known miRNA; **(B)**: novel miRNA. X-axis represent the fold change values between the two samples, the expression of wild type sample divided by the processing sample, Y-axis represent statistical test value (FDR), the higher represent the more significant differences. Two coordinate values are subjected to log treatment. Each dot represents a particular miRNA, the red dot indicates significantly up-regulated miRNAs, the blue dot represents a significant down-regulated miRNAs, the black dots represent non-significant differences miRNAs. The dot in the left represent down-regulated miRNAs, the dot in the right represent up-regulated miRNAs.
