# Supplementary material for: Integration of RNAi and RNA-seq Reveals the Immune Responses of Epinephelus coioides to sigX Gene of Pseudomonas plecoglossicida
Source: Front Immunol. 2018 Jul 16;9:1624. doi: 10.3389/fimmu.2018.01624 (PMC6054955; doi:10.3389/fimmu.2018.01624)
Supplement: Supplementary file 6 [file Image_6.PDF]

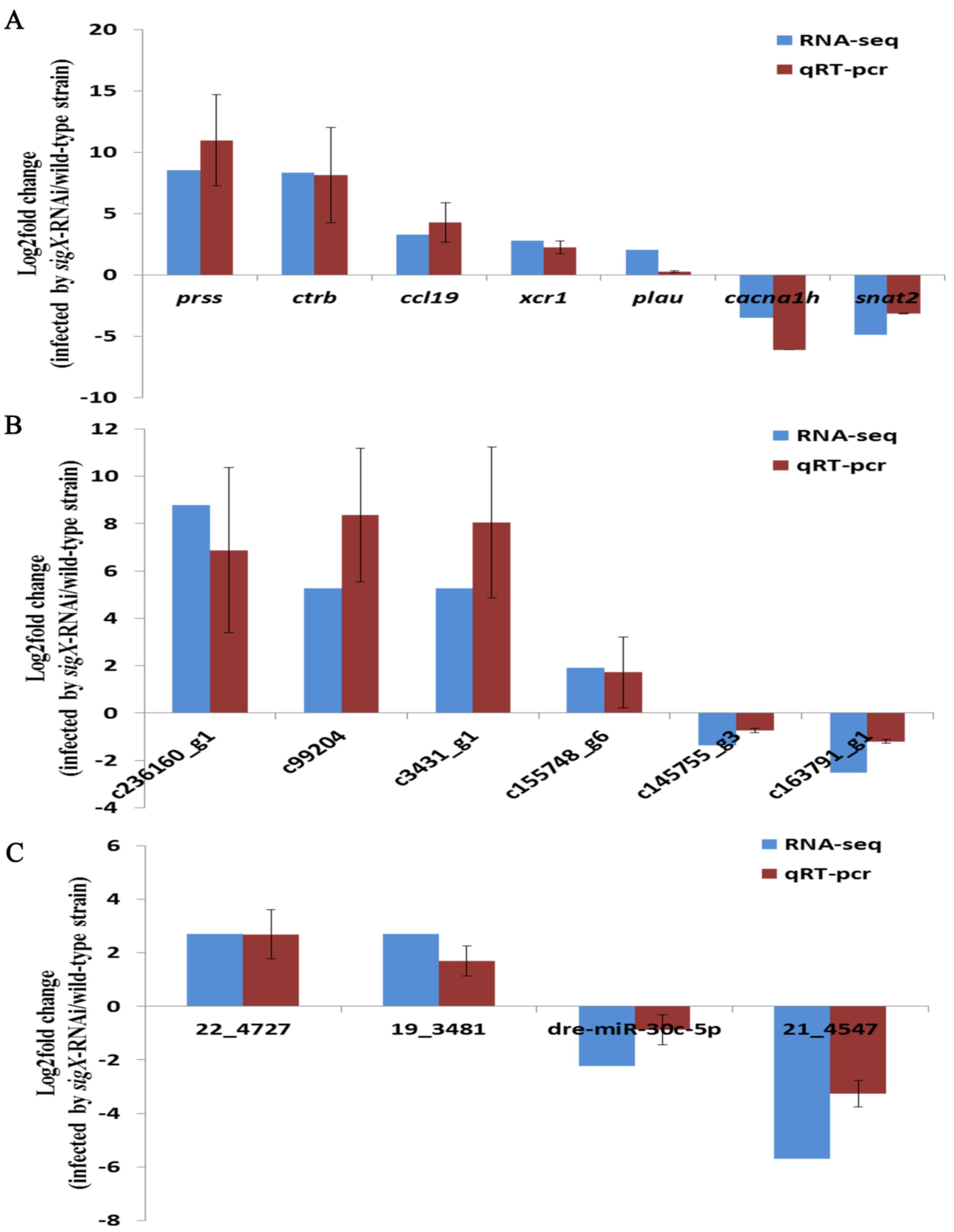

Supplementary Figure 6 verification of transcriptome data by qRT-PCR.

Relative changes in abundance of selected **(A)** mRNA, **(B)** lncRNA **(C)** miRNA. qRT-PCR was performed by triple technical repetions. Red bars: qRT-PCR; blue bars: RNA-seq.
