# Supplementary material for: Integration of RNAi and RNA-seq Reveals the Immune Responses of Epinephelus coioides to sigX Gene of Pseudomonas plecoglossicida
Source: Front Immunol. 2018 Jul 16;9:1624. doi: 10.3389/fimmu.2018.01624 (PMC6054955; doi:10.3389/fimmu.2018.01624)
Supplement: Supplementary file 7 [file Table_1.docx]

**Table S1** List of shRNA sequence used to silence *sigX* expression in present study

| **Name** | **Sequence(5’-3’ orientation)** |
| --- | --- |
| shRNA-216 | TGGGCAAGTCCAAGTTCAAGACTTCAAGAGAGTCTTGAACTTGGACTTGCCCTTTTTTT  GTACAAAAAAAGGGCAAGTCCAAGTTCAAGACTCTCTTGAAGTCTTGAACTTGGACTTGCCCATGCA |
| shRNA-239 | TGGCTCTACAGCATCACCTACATTCAAGAGATGTAGGTGATGCTGTAGAGCCTTTTTTT  GTACAAAAAAAGGCTCTACAGCATCACCTACATCTCTTGAATGTAGGTGATGCTGTAGAGCCATGCA |
| shRNA-240 | TGCTCTACAGCATCACCTACAATTCAAGAGATTGTAGGTGATGCTGTAGAGCTTTTTTT  GTACAAAAAAAGCTCTACAGCATCACCTACAATCTCTTGAATTGTAGGTGATGCTGTAGAGCATGCA |
| shRNA-396 | TGGTGCATGTGAACCCGATTGATTCAAGAGATCAATCGGGTTCACATGCACCTTTTTTT  GTACAAAAAAAGGTGCATGTGAACCCGATTGATCTCTTGAATCAATCGGGTTCACATGCACCATGCA |
| shRNA-405 | TGAACCCGATTGACCGGGAAATTTCAAGAGAATTTCCCGGTCAATCGGGTTCTTTTTTT  GTACAAAAAAAGAACCCGATTGACCGGGAAATTCTCTTGAAATTTCCCGGTCAATCGGGTTCATGCA |
