# Supplementary material for: Integration of RNAi and RNA-seq Reveals the Immune Responses of Epinephelus coioides to sigX Gene of Pseudomonas plecoglossicida
Source: Front Immunol. 2018 Jul 16;9:1624. doi: 10.3389/fimmu.2018.01624 (PMC6054955; doi:10.3389/fimmu.2018.01624)
Supplement: Supplementary file 8 [file Table_2.docx]

**Table S2** List of qRT-PCR primers used to determine gene expression changes in present study

| **Gene**  **type** | **Gene name** | **Forward primer sequence(5’-3’ orientation)** | **Reverse primer sequence**  **sequence(5’-3’ orientation)** | **source** |
| --- | --- | --- | --- | --- |
| mRNA | *sigX* | GAAGACAAGGCTCCGAAACCG | GCGACAAATCGAAGCACCAGAAT | This study |
| mRNA | *gyrB* | TGCTGAAGGACGAGCGTTCG | ATCATCTTGCCGACAACAGC | (1) |
| mRNA | *16S rDNA* | TCAGTATCAGTCCAGGTGGTCGC | CGTTACCGACAGAATAAGCACCG | This study |
| mRNA | *prss* | CGGGCGTTTACACCAAAGTCTG | TCTCCCGCTGCTTTTCTGTCTG | This study |
| mRNA | *ctrb* | AACACGTCTGTCGCTGTCACTTGT | TTTGTCAGTTCTTTTGTGGGTGGA | This study |
| mRNA | *xcr1* | CATGTCTGCGCTGGTTCTGTGA | GGTGCTGCTCGCTGTTCTTCTC | This study |
| mRNA | *plau* | ACAATCCTCCCTGCCCTTCG | AACCTGTTAGTCATCCTCGCCATT | This study |
| mRNA | *ccl19* | TTCTGTTCAGAACATCAGAACGGC | CTGTGGGCAAGTCAAGGCAAAT | This study |
| mRNA | *snat2* | AGAAAGCGAGAAACGGTGACAGA | ACCATTTGGTGGGTGAATGTTTG | This study |
| mRNA | *cacna1h* | AACTCCACTGTGGTGGTTTTGACC | TGTCCGCAGGGCTGGAACAT | This study |
| mRNA | *β-actin* | GGCTACTCCTTCACCACCACA | GGGCAACGGAACCTCTCAT | (2) |
| lncRNA | *c145755_g3* | GCTGCTAACTATGGTGGCTGGTG | GGTGCTTGTTGAGGTTGCTTGG | This study |
| lncRNA | *c163791_g1* | GCTCCTCAATTCCTTCAGTGTTCG | CACCGTAGTTAGCTGTCCCTCCAT | This study |
| lncRNA | *c155748_g6* | GCACATCAGCTTCTGCCCTCA | GCAACCTCACCACTAAATGCCACT | This study |
| lncRNA | *c236160_g1* | GAAGGGAGCCACTGAAGGTGTTG | GCTGATGGCAGGAGATGAAATTCA | This study |
| lncRNA | *c99204_g1* | CCCTGGGCGATACTCAAACTGTA | GCGCTTGCTTATGGAATGGC | This study |
| lncRNA | *c3431_g1* | CTCTTGCCATGAGCAGATGTTGAA | CTCCCCTCGTCTCCAGATGTTTTA | This study |
| miRNA | *5S rRNA* | GGAGACCGCCTGGGAATA |  | (3) |
| miRNA | *21_4547* | GGGCAGAGTGTGTCTGAAGCT |  | This study |
| miRNA | *23_4964* | GCCCAATCTGATGTCTGGTCCTT |  | This study |
| miRNA | *dre-miR-30c-5p* | GCCCTGTAAACATCCTACACTCTCAG |  | This study |
| miRNA | *22_4727* | GGTCACAGTGAACCGGTCTCTTT |  | This study |

**Supplementary references**

1. Izumi, S., Yamamoto, M., Suzuki, K., Shimizu, A., & Aranishi, F. Identification and detection of *Pseudomonas plecoglossicida* isolates with PCR primers targeting the *gyrB* region. *Journal of Fish Diseases* (2007) 30(7):391–397. **DOI:** 10.1111/j.1365-2761.2007.00820.x
2. Kuan, Y. C., Sheu, F., Lee, G. C., Tsai, M. W., Hung, C. L., & Nan, F. H. Administration of recombinant Reishi immunomodulatory protein (rLZ-8) diet enhances innate immune responses and elicits protection against nervous necrosis virus in grouper *Epinephelus coioides*. *Fish Shellfish Immunol* (2012) 32(6):986-993. DOI: 10.1016/j.fsi.2012.02.008

3. Chen, W., Yi, L., Feng, S., Zhao, L., Li, J., Zhou, M., ... & Lin, L. Characterization of microRNAs in orange-spotted grouper (Epinephelus coioides) fin cells upon red-spotted grouper nervous necrosis virus infection. Fish & shellfish immunology(2017) 63, 228-236. doi: 10.1016/j.fsi.2017.02.031
